# Chicago Medical Society

**Published:** 1886-08

**Authors:** 


### CHICAGO MEDICAL SOCIETY.

*Meeting, June 7, 1886.*—THE PRESIDENT, E. J. DOERING, M. D., in the chair.

PROFESSOR A. REEVES JACKSON read a paper entitled

THE INTRA-UTERINE STEM IN THE TREATMENT OF FLEXIONS, exhibiting the stems used. The essayist began treating uterine flexions with the stem pessary in 1870. Prior to that time the only methods he had employed were gradual dilatation and incisions. The results were so unsatisfactory that he sought for a safer and more successful method. Having received the impression that the use of the stem pessary was more hazardous than either the dilating or cutting plans, he commenced its employment with misgiving, and did not rely wholly upon it, but preceded it with either gradual stretching or slight incisions. In two cases this mixed method was followed by pelvic abscess, a sequence which he had never observed when the stem alone had been used. All cases of uterine flexion are not accompanied by dysmenorrhœa or sterility, yet when there exists a relationship between these symptoms and an existing flexion the latter must be looked upon as a mischievous factor and one that should be removed. He had never treated any case of flexion in which dysmenorrhœa was not present, although coexistent barrenness has been frequently an additional incentive to the patient to undergo efforts at cure.

He preferred Chambers' bifurcated vulcanite instrument, although the divergence of the branches *below* the internal os uteri was a radical defect in the instrument as ordinarily used. Frequently the branches should be closed so that the stem might be practically single in that portion which traverses the cervix. His method is as follows : A flexion and its direction being diagnosticated, a flexible bougie is passed through the bent portion of the canal and quite to the fundus. The depth of the canal being carefully noted, a pliable stem, consisting of the distal portion of the same, or a similar bougie, one-third of an inch shorter than the ascertained depth of the canal, is selected for introduction. A flange or bulb is formed upon the outer end of the stem by rolling upon it a section of rubber tubing. The woman being placed on the back, in Simon's position, and the os uteri exposed with a speculum, the stem, either grasped with a dressing forceps or mounted upon the end of a piece of pointed wire, is passed entirely into the uterus. A large tampon of cotton moistened with slightly alumized glycerine is pressed against the bulb of the stem, and allowed to remain one or two days. The tampon is removed and replaced at suitable intervals, until the tendency of the stem to leave its position disappears. After this yielding stem has remained from one to three weeks, according to the degree of tolerance manifested by the uterus, it is removed and a thicker one put in its place. This likewise is permitted to remain a week or two, and is then replaced by a Chambers stem. While not very much, or, indeed, any change of shape is to be expected in consequence of the use of the flexible stem, yet, in several instances, a very considerable alteration took place within a few weeks, or even a few days, and in a few cases it was found unnecessary to resort to a rigid instrument at all. Usually, however, it had been necessary to use an inflexible instrument for from three months to a year—not continuously, but for periods of three or four months, with an interval of a week or two, during which the stem was removed in order to test the degree and permanence of the improvement. The feature of this treatment which is essential to its safety and success is its slow and gradual conduct, and the non-observance of this necessity has been the cause of dangerous results and failures to cure.

The drawbacks attending this method of treatment were: 1. Difficulty in retaining the instrument in position; 2. Pain; 3. Hæmorrhage; 4. Pelvic inflammation—all except the first being common to all other methods of treatment. A table comprising the details of sixty-four cases treated by the intra-uterine stem alone was given, showing the ages and social conditions of the patients, the direction of the flexion and the result of the treatment. Of the entire number, 42 occurred in married and 22 in single women. Of the former, 8 had borne children; the other 34 were sterile. Of the latter, 8 subsequently bore children. A cure of the flexion followed in 40; of the remaining 24, four were improved and relieved of dysmenorrhœa. In 20 the result was unknown. The ages of the patients ranged from 19 to 39 years. The uterus was anteflexed in 50 and retroflexed in 14.

In conclusion, the author said: "I believe the principle of the intra-uterine stem in the treatment of flexions to be correct; and it need not be dangerous—at least, no more dangerous than any other effective method. I further believe that by its use more cases of uterine flexion can be cured than by any other means at present in vogue. The conditions of both safety and success are watchfulness, patience and slow progress."

PROFESSOR D. T. NELSON, in opening the discussion, said: Mr. President, I am glad to have heard the paper, and think it is a most valuable one. The cautions that it gives are certainly those that all of us should remember, to wit: the length of the instrument used compared with the length of the uterus, the slow and gradual dilatation of the uterus before using the inflexible stem, and removing it on the occurrence of bad symptoms. In recent years I have not been in the habit of using the stem pessary as much as my friend Professor Jackson, but I think that with his present instructions I shall try it again. Not that I have not tried gradual dilatation, and the gradual, slow, careful straightening of the uterus, but I have not by this particular means caused the pessary to be retained as constantly as he has. The vulcanite pessary, and the various other forms, including the Wright or Chambers modification, I have used, and with many of the difficulties the doctor has narrated. But with his modification it seems to me very likely we can use them with better success. The irritation produced by them has been a great drawback, and in recent years it has been my habit rather to use the form of pessary recommended by one of our members, Professor W. H. Byford, the slippery elm bougie. It produces a gradual dilatation of the uterus, and often produces remarkable results in the treatment of the flexions, and I have had no bad results from its use. One point that the doctor did not emphasize sufficiently is that the instrument should not be retained long if it produces pain, but it should be removed and the patient put in bed. I should have preferred to have him give directions for the patient to remove the instrument if the pain continued for a long time; for if it does, the instrument ought to be removed, and if he should happen to be out of the city and the patient should be unwilling for anyone else to see her, serious disease might commence before he returned and removed the instrument. For this reason it is, and always has been, my plan to have the instrument so arranged, by a string or something of that sort, that the patient can remove it herself. We should remember that the instrument should be less than the uterus by a third of an inch; that the uterus is to be put into its proper shape, in a splint, as it were, and then expected to grow right; that it is not cured when it is straightened; if it has been displaced for a considerable time there has been an atrophy of the uterine tissue on one side, and it may take weeks, or perhaps months, to alter the nutrition of the different parts of the organ, and until that change has taken place it is not likely that the patient is permanently cured, unless pregnancy has taken place and altered the nutrition of the parts. As to pelvic inflammation, the author has been more fortunate than most of us in the use of stem instruments. One point I wish to add, viz.: That when there is any possibility of gonorrhœal poison lurking in the genital passages of the female, greater care should be taken in the use of such instruments, or operative procedure of any sort, for that matter. I feel, when there is reason to suspect that this poison has once been implanted, that I hardly dare to introduce sound, pessary or other instrument in the interior of the uterus, and believe that such an instrument should be used with the greatest caution in these cases.

PROFESSOR E. C. DUDLEY said: Mr. President: The marvelous freedom from dangerous inflammation following the treatment of uterine flexure by forcible dilatation and by the application of the intra-uterine stem, furnishes a striking illustration of the fact that the human uterus will sometimes endure an immense amount of abuse. My own preference is generally for the former method, as advocated by Goodell,

Ellinger and others. My experience has only tended to confirm me in the impression that forcible dilatation is reasonably satisfactory in its results, and that the results are resonably permanent. I would seldom advocate the use of intra-uterine stem pessaries for retroflexion unless the flexure were of the so-called congenital variety, and therefore associated with atrophy of the uterus, a condition which is very rare. The essayist has, perhaps for reasons of brevity, omitted to make the distinction between physiological and pathological anteflexion. This distinction within a few years has been quite clearly defined by Schultze, Fritsche and others, and their teachings are now recognized as correct by many of the leading gynæcologists throughout the world. In the light of their investigations the old diagram of Kolrausch, which for more than twenty years has generally formed the basis for the illustrations of the normal position of the uterus, is now quite generally discarded. The uterus has no absolutely fixed position, but it has a certain normal range of movements. The angle between the body and the cervix may vary, according to the varying quantity of material in the rectum and bladder, from zero to at least 45°; Fritsche says 90°, and his observation is possibly within the physiological limits. When the bladder is full the uterus becomes straight and the angle of flexure disappears. When empty, the angle may measure from 45° to 90°, and yet not be pathological. It is, moreover, probable that a flexure of much less than 45° when the bladder is empty should be considered pathological. Furthermore, anteflexion, even within the defined limits, is always pathological if there be immobility at the angle of flexure; indeed, a displacement exists whenever the organ is restrained from its normal movements. In a word, anteflexion is pathological if the mobility at the angle of flexure be increased or decreased beyond the physiological limits, or absent. Want of a clear understanding of these simple facts has led to the invention of innumerable pessaries for straightening the anteflexed uterus, and they have been persistently employed, to the detriment of the patient, in cases of perfectly physiological anteflexion. Suppose a case: The uterus is shown by digital examination to be so low in the pelvis that when the bladder is empty its entire anterior wall is easily touched. The physiological flexure, which may be from 45° to 90°, is then perfectly apparent to the examining finger; the symptoms of vesical irritation are attributed to the flexure, and an anteflexion pessary is accordingly introduced which produces pressure upon the anterior wall of the uterus. The symptoms disappear, and the conclusion is erroneously formed that the relief was dependent upon the straightening of the uterus, when in reality the pessary has, perhaps, produced no such effect, but has merely lifted the uterus to its health level, and thereby relieved the symptoms, which were due not to flexure but to descent. The same manner of treatment has often been followed by relief from similar symptoms attributed to anteversion, when in reality the pessary, by lifting the cervix to a higher level, has exaggerated rather than reduced the anteversion. For this reason all vaginal pessaries especially designed for anterior displacements are in no respect superior to the ordinary Hodge pessary; indeed, they are objectional, because in overcoming the descent they press upon the uterine wall and thereby cause irritation of the organ. Anteflexion is only a symptom which may result from any one of a variety of widely different causes, such as adhesions, uterine fibroid, parametritis posterior, or failure of the puerile uterus to develop at puberty. It would indeed be irrational to attempt the relief of a symptom due to such diverse causes by any single plan of treatment. The essayist would not attempt to do this, but he has neglected to specify the particular flexures for the relief of which he deems the stem applicable. Inasmuch as many of these flexures are dependent upon the uterine or peri-uterine inflammation, and inasmuch as there is reason to conclude that dysmenorrhœa and other evils are more the result of the inflammatory state than of the flexure itself, I would advise that the stem be reserved for cases which are not relieved after the inflammation has been removed by safer methods. Such a plan would certainly restrict the use of the stem to a very small number of cases, because the symptoms for which it is to be employed would so often disappear upon the cure of the inflammation. It is indeed probable that the dysmenorrhœa for which the author has employed the intra-uterine stem may depend rather upon some faulty nutrition, or upon some disease of the uterus independent of the flexure, and that the stem therefore gives relief by some change which it produces in the nutrition of the organ. If this be true, it would then follow that anteflexion *per se* really furnishes no positive indication either in itself or in its results, but that the same treatment would be equally effective under similar conditions without the co-existing flexure. Congenital anteflexion of the puerile uterus is undoubtedly a condition for which the stem may be considered one of the legitimate means of treatment. Sterility, whether associated with pathological flexure or not, has been successfully treated by the stem. Winckel says that the presence of the instrument may give a better development to the menstrual decidua and thereby make a better bed for the ovum. One objection to the stem, strongly urged by Schultze, is that by its use the physiological flexure is overcome, and it therefore may be said to produce, rather than to relieve, displacement. But we should not permit theoretical considerations to bias our judgment in face of the author's carefully observed results. His contribution is certainly a valuable one, and shows that the instrument, at least in careful hands, is less dangerous than is ordinarily supposed. The author's freedom from inflammatory results is doubtless due to his judicious preparation of each case by means of the olive-tipped bougie. Undoubtedly the observations of Professor Jackson and others must be considered as placing the intra-uterine stem among the useful and approved resources in the treatment of these troublesome cases, but even at the risk of prolixity I again protest against the indiscriminate treatment of purely physiological anteflexion by any means soever.

DR. H. P. MERRIMAN said: I have very little to add to what has been said. The use of the various methods that have been proposed seem to me to aim at one given end,—to change the nutrition of the uterus. Forcible dilatation does that to a certain extent; it is temporary, however, in its action. Incision produces an alterative effect and accomplishes its purposes. It does not succeed a great many times, neither does the temporary action of dilatation. The use of the stem pessary, on the other hand, succeeds because it is keeping up a continuous pressure upon the parts. Now I am decidedly in favor of this treatment by stem pessary; it strikes me that it is the only rational method of treating these flexions, which are pathological. After the cause of a flexion has been removed,—that is, the inflammation of the uterus, or the pressure of a tumor, or pressure of heavy clothing, or whatsoever causes it,—the uterus does not always return to its natural state, and then we need to introduce some method for restoring it to its normal condition, and I do not know any more rational method than this one. This paper strikes me as a very valuable one. The valuable part of this treatment seems to me not to be so much in the use of this stiff stem as the earlier treatment by the flexible stem, where, by continuous pressure upon the parts, we are able to accomplish the same effect as passing a sound in chronic cases of gleet, producing a healthy action in a diseased organ and thus producing absorption of a pathological exudate. It strikes me that the doctor recognizes this condition, for often before using the stiff Chambers stem when he has been using these bougies, in a great many instances he has found the treatment has nearly cured the disease, and if it had been continued longer I believe a cure would have been effected. The intra-uterine stem, by continuous pressure, induces an alterative action of the tissues, the absorption of exudates and a gradual return to the normal condition of the uterus, and a natural tendency toward a straightening of the uterine canal as the uterus becomes healthy.

PROFESSOR SARAH H. STEVENSON said: I have listened to the paper with a great deal of interest, and also to the discussion. My methods are different. I have used the stem pessary a great deal in former years, but for the past two years I have discarded it entirely as some of the results were unfortunate, although I think I have never had any serious results from the use of the stem. I now use, and have for the past two years, the galvanic current entirely, and it is applicable to all cases, especially in those in which the stenosis is so great as not to admit the passage of the bougie. I have never found a case in which I could not use this method with satisfactory results.

DR. H. T. BYFORD said: I quite agree with Dr. Dudley in his trite but very true remark, "It is wonderful what an amount of abuse the uterus will stand," and I congratulate Dr. Jackson that he has discarded incision and dilatation in treating flexions. I also congratulate him upon his good success. I believe the mortality from this treatment—the treatment by the intra-uterine stem—has been estimated to be from ½ to 1 per cent. by those who have investigated heretofore. Whether it is so now I do not know. The present per cent. of inflammation of the cellular tissue varies from 2 to 5 per cent., as nearly as I can determine. There are an immense number of cases in which the stem caused inflammation, which have never been published. It seems to me that in considering this subject the reason for this treatment should be made more apparent. There are some who use it as a splint or merely to straighten the uterus; others use it as a stimulant on account of its continuous pressure. There is no doubt it stimulates and temporarily straightens the uterus, but it is well known that in time, in a large proportion of these cases, the uterus again becomes flexed. The question arises, should we try to straighten the uterus? As Drs. Dudley and Schultze have said certain flexions are supposed to be physiological (which I don't believe), the uterus is supported in the neighborhood of the internal os, which may be said to have a fixed place in the pelvis. The elasticity of the tissue will allow that part ot the uterus to be pressed in nearly all directions, but it will come back. The fundus may bend forward or backward and remain in such position for some time, and the uterus still be in a normal position. During youth the child who sits too much, has a curved spine, etc., having a uterus pretty firmly fixed at the cervix, will often have the uterus pressed upon by the abdominal contents in the wrong direction. The normal resistance of the uterus to flexure will be gradually overcome (the uterus may even become atrophied), and a flexion results which, when slight, may be called a physiological flexion, and may exist without causing trouble; but it is pathological. The elasticity which the uterus of normally firm structure displays during the filling up and emptying of the rectum and bladder is hardly worthy of the name of flexure. Any considerable permanent flexure occurring in this way must be the result of want of firmness in the structure of the uterus. If we are going to use a supporter we should use it when the flexure is forming, not after it has been produced. If we will use such treatment as will remove the improper pressure upon the uterus, viz., by straightening up the spine, using exercise, etc., etc., a stem will be seldom necessary, because whatever flexion has already been produced will usually not cause unpleasant symptoms. If it has gone to the degree of producing atrophy of the uterus we may need to use a stem pessary, but as a stimulant to the uterine tissue rather than a straightener of this organ. I have seen uteri bent almost like a horseshoe become impregnated and return almost to their former degree of flexion. The intra-uterine stem, in view of its slight action as a support and powerful action as a stimulant, and its notoriously bad record, should be the last resort. The frequency with which Winckel uses the stem is now about once in 218 cases, while formerly he used it about once in 50, and he is using it less all the time. In my experience and the experience of a great many others, if we cure the acute or subacute inflammation of the uterus and then apply stimulating measures we nearly always accomplish the cure of the flexion by safer means. There are, of course, a few cases left in which the use of the uterine stem may be justifiable, but I think they are exceedingly rare. If those present, following Winckel, use them only once in 218 times, but few of us will live long enough to do a great deal of harm.

PROFESSOR T. D. FITCH said: Mr. President: I think a paper so commendatory of a measure as this, will perhaps lead many of the members of the profession to adopt it without proper precautions and without realizing the dangers which attend the use of the intra-uterine stem. I believe it is a very dangerous instrument to use. I am an advocate, as you all know, of pessaries, but I do think the intra-uterine stem a dangerous instrument, and that in less careful hands than Dr. Jackson's serious results will often follow. My own experience in the use of it has been limited, for the reason that I became alarmed from the bad reports of cases by Dr. Chambers himself, the inventor of this bifurcated instrument which Dr. Jackson has exhibited. If the same precautions are used that are advised by Dr. Jackson, I think, as a rule, it might be entirely harmless; no, I should hardly be able to say entirely harmless, or entirely free from danger, but I think the precautions which he has adopted have been very ingenious and would in the majority of cases prevent serious results from the intra-uterine stem. His use of the bougies preceding the use of the inelastic stem, accustoming the uterine mucous membrane, or the uterus itself, to the presence of a foreign body within its cavity, is very ingenious, and a thing I should never have thought of myself. Although I have tried these pessaries occasionally, my great difficulty has been to keep them in the uterus; I might open them in any direction I pleased, spread the blades as widely as I pleased, and they would slip out—

they caused so much uterine contraction that they would be expelled from the uterine cavity into the vagina, and I have always been disappointed in the results from their use. For several years while I was in active practice I had adopted the treatment of Peasley for flexion and stenosis, whether caused by exudation or spasmodic contraction of the os internum; that is, by the use of his uterotome dividing the stricture at the internal os, and then gradually dilate the canal until I could introduce a No. 12 or 14 sound through the os internum. This was introduced every second day from one week to two weeks, until it ceased to be followed by pain and by hæmorrhage after its introduction, showing that the os internum had been thoroughly dilated and the incision had healed sufficiently so that no blood followed the use of the sound. After the sound was introduced I used a large glycerine tampon for the purpose of depletion and relief from irritation and to support the uterus; if it was an inversion it would hold the fundus up so as to assist in relieving the flexion to a certain extent at least, and preventing the occurrence of inflammation. I have treated a great many cases in this way and with entire satisfaction, and never had a case of acute inflammation of any kind occur as the result. I believe, however, that a majority of cases of flexion are attended with versions more or less. I don't believe that flexions occur so frequently as is generally supposed, unaccompanied with version; the uterus is tipped over more or less in connection with the flexion, and in connection with the treatment which I have suggested I have always corrected the version, and used the ordinary support or pessary to keep the uterus in its proper place, thereby relieving any contraction or pressure which would keep up the flexion. I think the paper an admirable one, and the Doctor's precautions in the use of the instrument he has advised commendable.

DR. H. C. FEDER said : I would like to ask for information. In the report of these sixty cases of inversion and retroversion it has not been stated how many were accompanied by prolapsis, or what was the cause of flexion ; whether in married women getting up too soon after confinement, or whether from acute inflammation. The paper does not go into the facts and state whether the uterus is lightened and thereby goes back of itself to a normal position, nor does it inform us if this could be assisted by giving medicine internally. Much depends upon the state of the patients at the time of treatment,—whether they are in a healthy condition, or whether they have some specific blood disease in which medicine would assist in the treatment. And if the medicine has an alterative effect, how much benefit is received from the medicine and how much from the pessary.

PROFESSOR JACKSON said, in concluding the discussion : Mr. President : I feel that I ought to express my thanks for the courtesy with which this paper has been received. It is only a thirteen minute paper, and there are a great many things in the domain of medicine that are not in it, and a great many questions might be asked on subjects growing out of and connected with it which I could not answer if I were disposed to. The intention of the paper is simply to demonstrate the efficacy of a single remedy in correcting a single deformity. Questions as to whether the uterus was prolapsed, whether the patients had taken antibilious pills, or had cachexia, really do not enter into the consideration of the subject. I supposed that was perfectly plain from the fact that no mention was made of anything beyond the mere condition of deformity. I am very glad so many excellent ideas have been added to it. The suggestion of Dr. Nelson as to the patient being able to withdraw the pessary is excellent, and is never omitted. I never introduce a pessary that I do not attach to it a silken cord by means of which the patient can withdraw it in case of necessity. As a rule, every patient should be able to withdraw any instrument placed in the genital passages; the regular attendant may not be at hand when needed, there may be an aversion on the part of the patient to calling in another physician, and she should have the proper means at her disposal. The remarks of Dr. Dudley as regards the distinction that should be made between pathological and physiological conditions resulting in flexion are quite proper, and I agree with him fully. We all know that the conditions preceding and accompanying these bent conditions of the uterus are very various; and in many cases no stem, incision, or other means will have a beneficial effect, although they may for a time cause the uterus to be straight. But the mere straightening is not always the main element of cure. When the uterus has been chronically fixed there will be a thinning of the side towards the angle, showing local failure of nutrition either as cause or effect of the bending. Straightening, therefore, is one element of cure in a uterus where there is insufficient nutrition, and I do not believe that any other means exclusive of this cures flexion. But it must not only be straightened, but its circulation must be fully restored, otherwise the organ will resume its bent condition. We cannot put a splint on the outside of the uterus, and the intra-uterine stem affords a means by which the uterus may be kept straight enough to allow of circulation on each side. The method of treating flexions by forcible and extensive dilatation does more than dilate. It straightens, also. A bend may be just as acute in a large tube as in a small one, and mere stretching will not suffice, and its results usually not be permanent. Gradual dilatation is much more promising, and next to the method by the stem I would prefer it. I have only treated cases of flexion in which dysmenorrhœa was present, a symptom that interferes with the patient's health, and the dysmenorrhœa was usually cured or relieved. I do not think this such wonderful success; only about two-thirds of the cases were cured, some were simply improved, and in some I do not know the result. Yet I think there is no other method that will do quite as well. The suggestions made in the discussion accounting for the safety and success of the treatment are, I think, correctly attributed to the preliminary measures—the slowness of the straightening and the promptness with which any tendency to harm could be met. The object was to accustom the uterus to its tenant, so that by and by it would accommodate a larger one, and in this way the uterus has been made to receive and tolerate the presence of an inflexible instrument. In one case a Chambers stem was retained twenty months, and I think if the patient had not returned and told me she was wearing it it would be there yet. It produced no unfavorable symptoms.

DR. A. R. SMALL reported

A CASE OF PISTOL-SHOT WOUND.

May 2d, 1886, he was called to see F. R., aged 23, who, a few minutes previously, had received a shot from a No. 32 pistol. Patient was suffering from shock, difficult breathing, and excessive pain in the left leg below the knee. The ball had struck the right eighth rib, about two inches external to the costal cartilage. Sensation was lost in the right leg below the knee. Motion was not impaired in the right leg, though the sensation was lost below the knee. The left leg was hyperæsthetic below the knee, and motion slightly impaired. A drainage-tube was inserted about two inches into the wound, and the wound dressed antiseptically. The patient complained of no pain except in left leg below the knee, where the pain was excessive. Morphia was given hypodermically in sufficient doses to control the pain. Nothing was allowed the patient the first twelve hours but ice, and occasionally water. About 10 P. M. there was evidence of internal hæmorrhage, and the patient seemed to be sinking. Milk was then given in small quantities frequently. The morning of the 3d he had rallied somewhat.

The urine was drawn by the catheter every eight hours, and contained blood. There was no expulsive force to the bladder. Respiration was normal after the first two hours.

On the afternoon of the 3d patient became delirious, and continued so, with occasional lucid intervals, until death, which occurred at 4.20 P. M. of May 4th.

Autopsy five hours after death. Rigor mortis well marked. Unfortunately, through a misunderstanding, the undertaker had preceded us and injected his preserving fluid, so that we were unable to determine exactly the amount of blood in the right pleural cavity. It must have been quite large, however, as the right lung was entirely collapsed. The ball made a clean round hole through the center of the eighth rib on the right side, about two inches from the costal cartilage, passed through the lower side of the right pleural cavity, without injuring the lung, passed through the diaphragm, right lobe of the liver, and superior portion of right kidney, and through the intervertebral foramen between the eleventh and twelfth dorsal vertebræ, on the right side of the spine, and lodged against the posterior surface of the body of the eleventh dorsal vertebra, just within the spinal cord, where it was so firmly imbedded that it could not be removed without disarticulating the spine, which, for sufficient reasons, we did not do.

Though we found the right lung collapsed, respiration had been normal except the first two hours after the injury.

DR. ALFRED S. HOUGHTON read a paper on

THE DANGER IN SPECIALISM.

He said that specialties had greatly increased medical knowledge and skill, and had secured for many much reputation. Hence young practitioners grasp any excuse for becoming specialists. But man is not a machine, but a complicated organism, and disease is complex, one organ sympathizing with another; hence it is necessary to examine every portion of the body and treat all organs affected. Hence there is danger in a specialist limiting his sphere of action and usefulness to an unnecessary degree. Another danger is that specialists are apt to become egotistic, and give rise to utterances which they will afterward regret.

